# The clinical implications of digital technology

**DOI:** 10.1177/13591045221145400

**Published:** 2022-12-16

**Authors:** Saam Idelji-Tehrani, Bernadka Dubicka, Richard Graham

**Affiliations:** 1 Department of Child and Adolescent Psychiatry, Institute of Child Health, 11700 University College London (UCL), UK; 2 Department of Child and Adolescent Psychiatry, Hull & York Medical School, 8748 University of York, UK; 3 Department of Child and Adolescent Psychiatry, 9022 Greater Manchester Mental Health Trust, UK; 4 Manchester Academic Health Science Centre (MAHSC), University of Manchester, UK; 5 Department of Child and Adolescent Psychiatry, Stem4, London, UK; 6 Keeping Well Southeast London Community, UK; 7 Digital Well-Being Consultant to Own it App Project

**Keywords:** CAMHS, internet, social media, dark web, mental health, children, adolescent, psychiatry, digital technology

## Abstract

The proliferation of digital technology within the lives of children and young people (CYP) provides arguably one of the most significant clinical and ethical paradigm shifts in Child and Adolescent Psychiatry. One can argue that mental health research has taken a myopic approach to understanding the interaction between young people’s technology use and their mental health. Mental health clinicians also need a better understanding of the digital lives of CYP and how technology may be supporting or harming their mental health. Within this paper, we argue that greater longitudinal research is required, particularly in vulnerable groups, and that there is an essential need for a standardised digital use assessment (DUA) tool, which assimilates CYP use of technology and their vulnerabilities/resilience to online risks. We subsequently offer a series of questions clinicians can use to explore technology use by CYP. Such an aide memoire may empower clinicians to have wider discussions around digital technology use with CYP, while also helping to develop appropriate safety and management plans.

## Introduction

The advancement of digital technologies, such as the Internet, has irrevocably altered the daily lives of young people. Self-expression, community and even love have their own digital footprint now. Those born after 1995 are amongst the first generation to have their development influenced by the Internet (Betz, 2019). In the United Kingdom, 60% of 8- to 11-year-olds and 97% of 12- to 15-year-olds were found to have their own mobile phone (Ofcom, 2022). Similarly, 45% of teens have reported being online almost continuously (Anderson & Jiang, 2018), with screen-time also increasing during the COVID-19 pandemic (Pandya & Lodha, 2021).

Research has highlighted the potential benefits of digital technologies including development of a social group and development of identity (Bolton et al., 2013; Rafla, Carson, & DeJong, 2014). Young people with a history of self-harm, for example, have been shown to use the Internet as part of help-seeking behaviours (Frost & Casey, 2016). Technology has also increasingly been used in clinical settings in the assessment and management of young people’s mental health (Hollis, 2022).

Yet amongst the many purported benefits of the digital technologies, emerging studies have demonstrated its negative impact on psychological well-being (McDool, Powell, Roberts, & Taylor, 2020), neuro-cognitive development (Firth et al., 2019; Tamana et al., 2019), depression and anxiety symptoms (Barry, Sidoti, Briggs, Reiter, & Lindsey, 2017; Boers, Afzali, Newton, & Conrod, 2019; Stiglic & Viner, 2019), and self-harm and suicidal ideation (Daine et al., 2013; Mars et al., 2015; Marchant et al., 2017; Rodway et al., 2022). Using general population data, Orben and Przybylski (2019) found a small but negative association between digital technology use and adolescent well-being. A recent update demonstrated that there may be windows of sensitivity to social media in adolescence, where higher social media use predicts lower life satisfaction. These windows occur at the age of 11-13 years for girls, 14-15 years for boys and 19 years for both boys and girls. (Orben, Przybylski, Blakemore, & Kievit, 2022). This study is reflective of the complexity seen in vulnerable adolescents in clinical practice and marks a possible transition away from the unhelpful dichotomy of whether social media is harmful or not.

However, fervent debate remains regarding the level of impact of digital technologies on child and adolescent mental health. In fact, in the aforementioned 2019 paper, Orben and Przybylski (2019) concluded that the association is so minimal that it does not have wider practicality for practice or policy and compared the negative impact to that of eating potatoes. Furthermore, Odgers and Jensen (2020) demonstrated no evidence to support causal claims related to smartphones, social media and adolescent mental health.

Nevertheless, 32% of 17–19-year-olds have reported that the Internet has a negative impact on their mental health (Nominet, 2021), and there is burgeoning research on vulnerable groups and a spectrum of online risk beyond simply the amount of time spent on screens (El Asam & Katz, 2018). Children and young people (CYP) who are particularly vulnerable online include those with family difficulties, disabilities, mental health difficulties, emotional/behavioural difficulties, and neurodevelopment disorders; as well as marginalised and disadvantaged groups (including children involved in gangs and young carers) (Davidson, Aiken, Gekoski, Phillips, & Farr, 2021; Davidson, Bogaerts, Caretti, & Aiken, 2016; Katz & El-Asam, 2020; Livingstone & Palmer, 2012; Livingstone et al., 2017).

## Are We Suffering From Digital Myopia?

It has been argued that a myopic view has been applied to mental health research related to digital technologies and its risks. Research has primarily focused on screen time in the general population, gaming and Internet addiction, with other areas neglected (Aboujaoude & Gega, 2021).

Whilst clinicians must be aware of Gaming Disorder (now classified in DSM-V) and accounts of Internet Addiction, a limited focus blurs the wider clinical and safeguarding perspectives presented by digital technologies and their associated risks. Some of these risks include: exposure to online hate; sexual exploitation; and exposure to suicide-related content. Additionally, social media allows for unrealistic social or body comparisons (Aref-Adib et al., 2020; Mahon & Hevey, 2021), as well as the quantification of social acceptance through ‘likes’ and ‘followers’ (Diefenbach & Anders, 2022). These comparisons can impact on self-esteem and foster negative self-perceptions, which can impact the mental health of CYP (Firth et al., 2019).

## Digital Exclusion

Digital exclusion can be defined as the inability to access Web-based services. Material deprivation, old age, severe mental illness and social isolation have all been highlighted as risk factors for digital exclusion (Greer et al., 2019; Spanakis, Peckham, Mathers, Shiers, & Gilbody, 2021).

Although digital exclusion as a phenomenon was present prior to the COVID-19 pandemic, the pandemic marked a significant shift of life and services to the online space. This shift has consequently increased digital exclusion (Holmes & Burgess, 2020) and heightened inequalities in educational and occupational prospects (Good Things Foundation, 2021; Honeyman, Maguire, Evans, & Davies, 2020; Watts, 2020).

Clinicians must be aware of this growing digital chasm, as it could affect CYP’s educational attainment, self-esteem, and development of skills necessary for a rapidly evolving digital future.

## Need for More Sophisticated Research

Research needs to move away from cross-sectional data of general population samples, which rely on self-report measures of screen time. Longitudinal research is required with CYP at different developmental stages, examining the benefits and harms of different types of interactions with technology. Crucially, research must consider the content, context and impact on vulnerable groups. Clinicians should be aware that certain groups appear to be more at risk online, with these groups either encountering risk online more often, or finding risky content more harmful (Dubicka & Theodosiou, 2020; El Asam & Katz, 2018; Livingstone et al., 2017).

For example, the use and perceived negative impact of social media is greater in 11–19-year-olds with a mental health disorder (NHS Digital, 2018). Despite these vulnerabilities, research on technology use has focused on general population samples, where the effects on these sub-groups are likely to be masked.

## A Digitally Informed Risk Assessment

With the proliferation of digital technologies in the lives of CYP, clinicians face a challenge in keeping abreast of their digital technology use. We would argue that clinical risk assessments should include a tailored digital use assessment. Any such assessment should not only be focused on the content consumed but also the context, especially with groups who may be more vulnerable to online risks or have minimal support or supervision.

Davidson (2021) devised a useful taxonomy of the risks and harms posed by digital technologies. Similarly, Livingstone and Stoilova (2021) have proposed the ‘CO:RE 4Cs’ of online risks. Such taxonomies can be harnessed to form a clinical aide memoire, allowing clinicians to cover an array of digital risks and protective factors. The Samaritans (2021) initiated the process of developing a clinical aide, focusing primarily on reviewing Internet use around suicide and self-harm. However, there is limited research on the use, risks and benefits of such an aide memoire or digital risk assessment (Aref-Adib et al., 2020).

Clinicians, such as psychiatry trainees, have little confidence in assessing digital risk (Aref-Adib et al., 2020), and will often utilise general risk assessment pro formas, which seldom contain prompts regarding Internet use. We have therefore delineated some areas that would be important to consider as part of a malleable Digital Use Assessment (DUA). Any such assessment would need to consider the age and developmental stage of the child.

## Digital Use Assessment

### Engaging CYP to Discuss Digital Technology Use

Questions should — like any clinical history — be open in nature and focus on engaging CYP to explain and consider their digital activities. This will not only allow for the development of rapport but also help guide questions around motivation and content engagement, for both helpful or potentially harmful use. Detailed questions can be asked if particular issues arise. For example, if a young person is self-harming, questions about online activities relating to self-harm and suicide should be explored. Similarly, questions around sources of support for self-harm are also important.

Initial questions should cover what young people do online, as well as when, with whom and for how long. If concerns arise around digital use, it may be helpful for the young person to consider keeping a ‘digital diary’, describing their Internet use from the start to the end of the day.

Table 1 offers a list of potential questions clinicians could utilise to explore a CYP’s online life, depending on the clinical assessment and developmental age.

**Table 1.** The Digital Use Assessment (DUA) *Proposed questions to ask if clinically indicated

**Opening questions**
Tell me a bit about your online life
What do you enjoy doing online?
Do you use social media?
What other online platforms do you like using?
What apps do you use?
Do you enjoy gaming?
How much time do you spend online per day?
**Impact on health and well-being**
Do you feel that your use of technology has ever impacted your mood?
Does your use of technology ever impact your self-esteem?
Do you ever get FOMO (Fear of Missing out) from your online activity? How does this affect you?
Have you ever lied online in order to improve your social appearance?
Has your technology use stopped you from doing other activities?
Does your technology use impact your sleep?
**Further probes to consider**
Has anyone ever commented on your technology use?
Do you know anyone who has made any money online?
Do you make money online?
How do you spend money online?
Have you ever lost money online? If so, how and how much?
Has anyone ever shared anything online about you that you would consider to be ‘private’ without your consent?
Have you ever used the dark web?
**Aggression, hate and radicalization**
Do you ever see stressful or hateful things happening online?
Have you ever been bullied online?
Have you ever thought you might have bullied someone online?
Have you ever agreed with, or expressed/shared, views online that others have thought are extreme or dangerous?
Has anyone ever knowingly shared any content with you online that is possibly harmful to others?
**Misinformation**
What online information sources do you trust?
Do you ever fact check things you read on the internet?
How do you work out what is fact or fake online?
**Digital skills and resilience**
What offline activities do you enjoy? Would you like to do them more often?
What do you do when you see something hateful or stressful online?
What helps you recover if you see something hateful or stressful online?
How do you avoid upsetting content?
Have you thought about how you could get support if something uncomfortable was happening?
Do you ever go online to get support when you are upset?
Suicide and self-harm*
Do you ever see any content where people harm themselves? Did you purposefully search for this or was it ‘suggested for you’ by a website/app?
Do you ever read blogs/posts or watch videos from people who are experiencing difficulties with self-harm or suicidal thoughts?
Do you look for materials to help you manage any thoughts of self-harm or suicide?
Have you ever used the internet to look for pro-suicide materials?
Have you researched what methods of suicide are lethal? Have you tried to find/buy any products to harm yourself?
Substance Misuse*
Have you ever bought illegal drugs online?
Have you ever sold illegal drugs online?
What platforms did you use to buy or sell illegal drugs? (e.g. social media, dark web)
Gaming and gambling*
How long do you game for?
Does gaming ever get in the way of normal daily activities?
Do you ever spend money in game, on skins or loot boxes? How much do you spend?
Do you ever watch others gamble online?
Do you use online casinos? How much do you spend?
Sexual risks*
What apps do you use for relationships?
Have you ever met anyone face to face that you met on the internet?
Do you message people generally your own age?
Have you ever had to deal with an adult who was interested in you?
Have you ever sent any sexual texts (‘sexts’)? Have you ever received any unwanted sexual texts?
Have you ever sent ‘nudes’ to anyone? Have you ever received any unwanted nudes from anyone? Has anyone sent nudes of you?
Have you ever been exposed to any sexual content that you did not want to see?
Have you ever been asked to engage in a sexual activity by anyone online?
Cyber-crime*
Have you ever been the victim of hacking?
Have you ever been asked to pay a ransom online?
Have you ever been the victim of fraud or identity theft online?
Have you ever been in trouble with the police for any of your online activity?
Have you ever been uncertain about the legality of your online activities?
Body image*
Have images or influencers made you feel less good about your body?
Have you ever seen any pro-anorexia content online?
Have you ever purposefully searched for pro-anorexia content online?

### Suicide and Self-Harm

The AVON Longitudinal Study of Parents and Children demonstrated that suicide and self-harm related internet use was prevalent amongst CYP who had self-harmed (Mars et al., 2015). Rodway et al. (2022) found suicide-related Internet experience in 24% of all young people aged 10–19 who died by suicide between 2014 and 2016. Search for information on suicide method was found to be the most common suicide-related Internet experience. Similarly, a systematic review conducted by Marchant et al. (2017) demonstrated a relationship between high levels of Internet use, searching for websites with self-harm/suicide content and self-harm/suicidal behaviour.

A bi-directional relationship between the Internet and CYP who self-harm has been found, with the Internet providing a valuable source of support (Marchant et al., 2017; Mars et al., 2015). Therefore, clinicians and researchers need to explore protective measures CYP take online. Similarly, it is important to explore how peer support groups may vary in terms of support or harm, depending on context, such as the young person’s mental state at the time.

Clinicians also need to be aware of access to the dark web, which may amplify risks. The dark web may offer a young person anonymity, access to more pro-suicide content (Morch et al., 2018) and access to more effective means of suicide (Sedgwick, Epstein, Dutta, & Ougrin, 2019). One could argue that by using the dark web, a young person is already showing both acts of potential planning and circumvention of detection, which will likely impact the perceived level of risk of death by suicide.

### Aggression, Hate and Radicalisation

It has been estimated that 19% of 10–15-year-olds in England and Wales experienced at least one type of online bullying behaviour in the year ending March 2020 (ONS, 2020). Data has also suggested that cyberbullying has remained constant during the COVID-19 pandemic (Vaillancourt et al., 2021). Yet cyberbullying may just be one facet of online hate and aggression encountered by CYP. They may also experience harassment or exposure to extremist content. Clinicians may also need to consider support for specific groups and their exposure to hate crimes online. For example, LGBTQIA + CYP may be exposed to homophobic and trans-exclusionary radical feminist (TERF) comments, with a rise in the incidence of both online and offline Anti-LGB and Transgender hate crimes being reported in the UK (Keighley, 2021).

### Substance Misuse

The dark web has been noted as being influential in the development of anonymous, online drug markets (Orsolini, Papanti, Corkery, & Schifano, 2017). A cross-sectional survey (Van Buskirk et al., 2016) demonstrated that dark web purchasers were more likely to be aged under 25. Furthermore, Barratt, Lenton, Maddox, and Allen (2016) hinted at the role of the dark web in accelerating drug use in older adolescents.

The rigmarole of accessing crypto-markets through the dark web may be prohibitive to young people. CYP may continue to access illicit substance and other psychotropic medications through surface web pages and applications, including WhatsApp and Wickr (Moyle, Childs, Coomber, & Barrett, 2019). Social media has made it easier for young people to buy drugs. A survey conducted of approximately 2000 16–24-year-olds found that almost one quarter reported seeing illicit drugs advertised for sale on social media (McCulloch & Furlong, 2019).

### Gaming and Gambling

A recent meta-analysis demonstrated a pooled prevalence of gaming disorder to be 4.6% (95% CI 3.4%–6.0%) in adolescents (Fam, 2018).

There has been growing concern regarding proliferation of ‘Loot Boxes’ within games and that it may be linked to gambling-related harm in both children and adults. In an attempt to monetise their product further, companies sell (for real-world money) additional in-game goods and ‘power-ups’. ‘Loot boxes’ are a purchasable collection of in-game items where the purchaser has no knowledge of what they will receive at the point of purchase. It has been suggested that loot boxes are psychologically akin to gambling. The UK Digital, Culture, Media and Sport Committee warned that loot boxes should be regulated under gambling law and not sold to children (DCMS, 2019). Zendle, Meyer, and Over (2019) demonstrated that loot boxes can cause problem gambling among older adolescents.

Alongside peer pressure and marketing, CYP may also be exposed to gambling through other sources such as Twitch and YouTube, where influencers stream themselves playing online casino games, and often promote sign-up links with monetary rewards. Consequences of problematic gambling include poor educational and occupational outcomes, interpersonal difficulties, and negative emotional states (Emond, Griffiths, & Hollen, 2022).

### Sexual Risks

The Internet Watch Foundation (2020) highlighted the growing risk children face online from sexual predators, with a sharp rise of 77% in self-generated images being sent in 2020 in comparison to 2019. Girls aged 11–13 were found to be particularly vulnerable. Child sexual abuse was recently highlighted by Akbarialiabad, Dalfardi, and Bastani (2020) as a specific area of concern for child psychiatrists to be aware of in relation to the dark web.

If concerns exist surrounding exploitation, clinicians should sensitively explore potential online sexual behaviours and abuse (Jonsson et al., 2019). Any screen of online sexual risks would likely include questions on sexting, cat-fishing (creation of a false identity online), self-generated naked photographs (‘nudes’), grooming, child sexual exploitation/coercion or child pornography. As with other forms of abuse, disclosure may prove to be difficult for CYP and it is more likely that online abuse may be discovered by someone else (Marie Collins Foundation, 2020).

### Misinformation

CYP are increasingly vulnerable to misinformation (Howard et al., 2021). 76% of 14–24-year-olds have reported seeing online misinformation at least once a week (Vodafone, 2020 cited in Unicef 2021). ‘Fake news’ can impact CYP’s mental health as well as skewing their world view (National Literacy Trust, 2018). Furthermore, misinformation may be traumatic to CYP and expose them to hateful content (CCDH, 2019). Clinicians may therefore wish to ask where CYP commonly receive information online and how they verify this information.

### Cyber-Crime

Within a forensic history, cyber-crime (hacking, identity theft, use of malware and cyber-fraud) should be considered. It is important to acknowledge that CYP may be both perpetrators and victims of online crime.

### Impact on Health and Functioning

The impact of digital technology use on health and functioning will be informed by the clinical assessment. Psychological well-being and mental health difficulties should be considered by clinicians working with CYP. Psychological processes such as loneliness and Fear of Missing Out (FOMO) may also be related to technology use (Barry et al., 2017; McDool et al., 2020).

Sleep is vital to the mental health of CYP (Shanahan et al., 2014), and can be negatively impacted by the use of screens (Lund, Sølvhøj, Danielsen, & Andersen, 2021). Lack of sleep can contribute to the onset, as well as persistence, of mental health disorders (Wang et al., 2016). Data from the CYPMH prevalence study during the pandemic showed that 69.6% of older adolescents (aged 17–22) with mental health problems suffered from sleep difficulties (NHS Digital, 2020).

Overuse of digital technology may also impact on physical activity and obesity. Children are becoming less physically active, which is partly driven by increasing access to digital technology (Whiting et al., 2021). Increased screen time has been associated with an elevated body mass index, increased adiposity, and unhealthy diet (Mitchell, Rodriguez, Schmitz, & Audrain-McGovern, 2013; Stiglic & Viner, 2019).

Understanding the impact of digital technology use and its sequelae on activities of daily living (ADLs), spending, education and social functioning (e.g., social withdrawal) will help characterise the level of impairment caused. As part of any assessment on impairment, clinicians should aim to understand whether digital exclusion has impacted on academic or occupational functioning.

### Digital Resilience

Resilience can be defined as a person’s ability to adapt to and manage challenging and stressful events. It is arguably impossible for parents and carers to monitor CYP’s Internet use and it is therefore essential to foster CYP’s ability to navigate the web safely and manage the plethora of possible risks independently within a variety of contexts (Byron Report, 2008).

Some key factors have been identified in facilitating the development of digital resilience. With suitable support and development of digital skills CYP have been shown to build digital resilience (Helsper, Schneider, Van Deursen, & Van Laar, 2020; Manning, 2021; Przybylski, Mishkin, Shotbolt, & Linington, 2014). An ability to self-regulate Internet use has been shown to build CYP’s resilience. Furthermore, social environment has been identified as being key in the development of CYP’s digital resilience. Parental guidance and encouragement of safe Internet use, rather than restriction, has been shown to be a crucial protective factor (Przybylski et al., 2014)**.** Supporting families and schools to help guide CYP’s digital technology use may serve as an important ongoing, preventative measure.

## Incorporating Digital Use Into Safety and Care Plans

If significant risks are disclosed, clinicians will need to follow local safeguarding policies. There may also be a need for clinicians to liaise with parents or other professional services, thus limits of confidentiality will need to be discussed from the outset of any assessment. In the UK, concerns about online child sexual abuse can also be reported to National Crime Agency - Child Exploitation and Online Protection (NCA-CEOP). Other information and support for parents and carers in the UK can be found at ThinkUKnow, NSPCC and Parents Protect. The Marie Collins Foundation offers support to CYP following online sexual abuse as well as support to parents and carers. Additionally, So You Got Naked Online has advice for young people (including CYP with special educational needs and disability) and parents affected by sexting.

There are a plethora of other general resources that can be provided to parents and carers in order to help keep children safe online. It may be worthwhile signposting parents and carers to organisations such as NSPCC, Childnet and Parent Info. Educate Against Hate Parents’ Hub provides support and resources to keep children safe from online extremism and radicalisation.

Care plans may need to consider incorporating other aspects of healthy technology use, for example when negotiating the impact on sleep routines and exercise. Care plans should also incorporate helpful technology use into CYP’s routines, such as online support and social networks.

Liaison with education may be helpful for those CYP who are struggling with digital exclusion.

**Figure 1.**
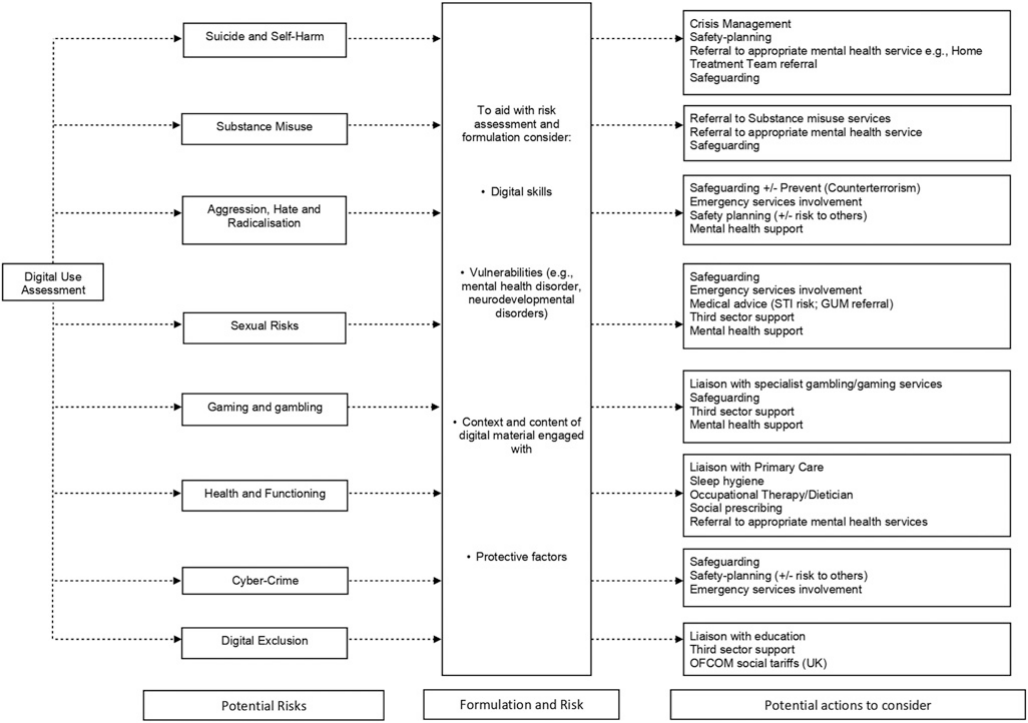
Flow-chart to demonstrate how the Digital Use Assessment (DUA) could be incorporated into care plans for CYP

Figure 1 presents a flow-chart of how the DUA can be incorporated into care plans for CYP.

## Conclusion

Clinicians working in Child and Adolescent Mental Health Services (CAMHS) need a better understanding of the digital lives of CYP and how technology may be supporting or harming the mental health of the children and young people they see. There is an essential need for the development of a standardised, balanced digital use assessment/aide for clinicians, which assimilates a detailed understanding of how CYP use technology, their vulnerability, as well as resilience, to risks. Such an aide memoire may empower clinicians to have wider discussions around digital technology use with CYP, while also helping to develop appropriate safety and management plans. Similarly, there is a need for sophisticated longitudinal studies examining the harms and benefits of digital technologies across a spectrum of use which may further inform clinical practice.
